# The Granulate Cutworm (Lepidoptera: Noctuidae): Biological Parameters Under Controlled Conditions, Host Plants, and Distribution in the Americas

**DOI:** 10.1093/jisesa/ieaa115

**Published:** 2020-11-07

**Authors:** Alexandre Specht, Fernando M S Dias, Germán San Blas, Vânia F Roque-Specht, Mirna M Casagrande, Olaf H H Mielke, Débora G Montezano, Izailda Barbosa Santos, Silvana V Paula-Moraes, Thomas E Hunt, Juaci V Malaquias, Felipe A D Bonfin, Paulo V M Vieira

**Affiliations:** 1 Embrapa Cerrados, Planaltina, DF, Brazil; 2 Departamento de Biologia Animal e Vegetal, Universidade Estadual de Londrina, Londrina, Paraná, Brazil; 3 Laboratório de Estudos de Lepidoptera Neotropical, Departamento de Zoologia, Universidade Federal do Paraná, Curitiba, Paraná, Brazil; 4 INCITAP-CONICET - Facultad de Ciencias Exactas y Naturales, Universidad Nacional de La Pampa, Santa Rosa, Argentina; 5 Universidade de Brasília, Faculdade UnB Planaltina, Área Universitária 1, Vila Nossa Senhora de Fátima, Planaltina, DF, Brazil; 6 Corteva Agrisciense, Marion IA; 7 Entomology & Nematology Department, West Florida Research and Education Center, University of Florida, Experiment Road, Jay, FL; 8 Haskell Agricultural Laboratory, Department of Entomology, University of Nebraska-Lincoln, Concord, NE; 9 Instituto Federal de Brasília, Campus Planaltina, Rodovia, Zona Rural, Planaltina, DF, Brazil; 10 Universidade de Brasília, Campos Universitário Darci Ribeiro, Instituto Central de Ciências, Asa Norte, Brasília, DF, Brazil

**Keywords:** biotic potential, immature development, life tables, pest management, reproductive biology

## Abstract

*Feltia subterranea* (Fabricius), commonly known as the granulate cutworm, is a common species of owlet moths (Noctuidae) of major agricultural importance, widely distributed in Nearctic and Neotropical regions. This study was conducted to determine the species biological parameters, gather information about its larval host plants, and assess the agricultural significance of this species in the Americas. The viability of the egg, larval, pupal stages, and prepupal period was 98, 98, and 100%, respectively, under laboratory conditions. The average duration of the egg, larval, pupal stages, and prepupal period was 3, 17, 4, and 13 d, respectively. All laboratory-reared larvae developed through five instars. The growth ratio was 1.93 for females and 1.85 for males. The duration of the larval stage was significantly longer in females than in males from the fourth instar. The duration of the pupal stage was significantly shorter in females than in males. When larval and pupal stage durations were combined, there were no significant differences in total development time as a function of sex. In total, 159 botanical taxa belonging to 41 families were recorded as host species for *F. subterranea.* The families with the greatest number of host species were Fabaceae (22), Poaceae (19), Asteraceae (16), Brassicaceae (13), Solanaceae (12), Amaranthaceae (7), Cucurbitaceae (7), and Malvaceae (5). It is noteworthy that the large number of native weeds used by *F. subterranea* as host plants could represent a significant source of infestation of crops in the agricultural landscape.

*Feltia subterranea* (Fabricius, 1794), commonly known as the granulate cutworm (Figs 1–9), is a common species of owlet moths (Noctuidae) of major agricultural importance, widely distributed in Nearctic and Neotropical regions (Lafontaine 2004) (Fig. 10). Early in its original description, the species was readily recognized by the subterranean habits and voracity of its larvae (Fabricius 1794). Even though authors recognized the presence of the species in Central and North America, *F. subterranea* is vaguely acknowledged as ‘America meridionalis’ in its original description (Fabricius 1794). The intraspecific variability of *F. subterranea* throughout its range of distribution can be inferred by the rather large number of species-level names, now recognized as synonyms, described from all through the Americas (Poole 1989, Lafontaine 2004): *Agrotis annexa*  Treitschke (1825) from North America, *Agrotis decernens*  Walker ([1857]) from Santo Domingo, Distrito Nacional, Dominican Republic, *Agrotis interferens*  Walker (1858) from Rio de Janeiro, Rio de Janeiro state, Brazil, *Xylina lytaea*  Druce (1889) from Xalapa, Veracruz, Mexico, and *Agrotis interposita* Maassen (in: Weymer and Maassen 1890) from Puracé, Cauca, Colombia.

**Figs. 1–5.**
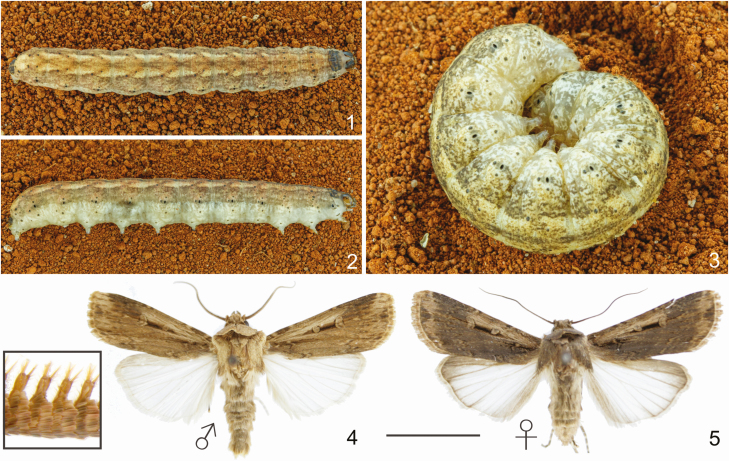
Habitus of *Feltia subterranea* (Fabricius, 1794). 1–3. Last instar larvae: 1. Dorsal. 2. Lateral. 3. Curled. 4–5. Adult: 4. Male (inset: detail of the antennae). 5. Female. Scale bar = 1 cm.

**Figs. 6–9.**
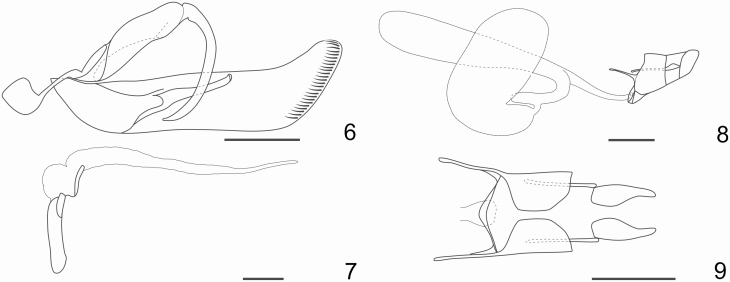
Male and female genitalia of *Feltia subterranea* (Fabricius, 1794). 6**–7**. Male genitalia. 6. Genital capsule with left valva and aedeagus removed, lateral. 7. Aedeagus with everted vesica, lateral. 8**–**9. Female genitalia. 8. Lateral. 9. Ventral: ductus, corpus, and appendix bursae hidden. Scale bar = 1mm.

**Figure 10.**
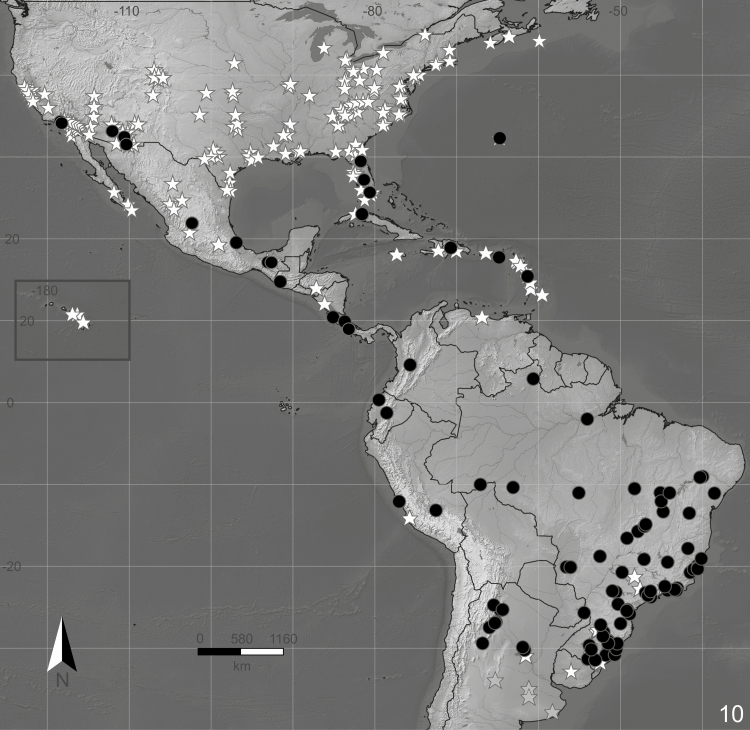
Distribution of *Feltia subterranea* (Fabricius, 1794). Black circles—data taken from examined specimens deposited in collections; white stars—data taken from literature; gray stars—uncertain records taken from literature.

The distribution of *F. subterranea* ranges from about the 40°N parallel in Nova Scotia, Canada (Ferguson 1953) to the 30°S parallel in Pelotas, Rio Grande do Sul, Brazil (Specht et al. 2004), including the Bermuda and Caribbean islands.

*Feltia subterranea* common names vary in English-, Portuguese-, and Spanish-speaking countries, but usually refers to the size of its larvae relative to other cutworms, the slow-moving behavior, subterranean habits, its typical curled posture (Fig. 3), or the leathery, granulated skin of the larvae (Figs. 1–3): ‘granulate cutworm’ (Lafontaine 2004), ‘subterranean dart’ (Wagner et al. 2011) in the United States and Canada, ‘lagarta-rosca’ (i.e., ‘curled caterpillar’) in Brazil (Silva et al. 1968), and ‘cortador pequeño’ (i.e., ‘small cutworm’), ‘gusano cuerudo’ (i.e., ‘thick-skinned caterpillar’), and ‘gusano cachazudo’ (i.e., ‘slowly caterpillar’) in Spanish-speaking Latin America (King and Saunders 1984, Coto et al. 1995, Lafontaine 2004). Although *F. subterranea* is widely known as an important pest of several crops in the Americas, the available information about its biology is patchy and occurs in several publications with different purposes, mainly focusing in North American populations. *Feltia subterranea* is still frequently misidentified in entomological collections and scientific publications with superficially similar species, especially in the southern part of its distribution, complicating the development of pest management strategies. At least three superficially similar species that until recently were recognized as synonyms of *F. subterranea*: *Agrotis anteposita* Guenée, 1852, *Agrotis blanchardii*  Berg, 1882 and *Noctua lutescens* Blanchard, 1952, replace *F. subterranea*, in Chile, Uruguay, and southern Argentina, respectively (Lafontaine 2004). In the referred countries, the distribution of these similar cutworms overlaps with that of *F. subterranea*, making misidentifications frequent (cf. Artigas 1974, Biezanko et al. 1974, Klein and Waterhouse 2000, Angulo et al. 2008, Dias et al. 2019).

The plant injuries caused by *F. subterranea* include mainly seedling stand reduction, defoliation, and fruit and stem boring. During the day, the larvae have the behavior to move beneath the soil surface, which provides shelter from natural enemies and foliar spray applications compromising the effectiveness of granulate cutworm management (Deitz et al. 1992).

Due to larval voracity, the economic impact of the species is acknowledged in several continents and countries, especially in North America (Howard 1897, 1900; Forbes 1903; Jones 1918; Crumb 1915, 1929, 1956; Whelan 1935; Chamberlin and Allen 1957; Eden et al. 1964; Lee and Bass 1969, 1970; Tietz 1972; Adlerz 1975; Deitz et al. 1992; Rings et al. 1992; Heppner 2007; Wagner et al. 2011; Prestes 2014; Capinera 2019), including Central America (Calderon 1931, King and Saunders 1984, Coto et al. 1995), the Caribbean islands, such as Puerto Rico (Wollcot 1941, 1948), and in several South American countries, such as Colombia (Gallego 1969, Posada Ochoa 1989), Venezuela (Guagliumi 1967), Peru (Valencia and Valdivia 1973), and Brazil (Fonseca 1934, 1937, 1939; Costa 1954, 1959; Mariconi 1954; Gallo and Flechman 1962; Silva et al. 1968; Zikán and Zikán 1968; Vendramim et al. 1982; Pereira et al. 2012).

For these reasons, the objectives of this study were to: 1) conduct life table studies under controlled conditions to describe the biology of *F. subterranea*; 2) compile host plant data from literature and new records through larvae collection conducted in agricultural regions in central and southern Brazil; and 3) compile the distribution of the species from literature and specimens deposited in entomological collections. In addition, illustrations of *F. subterranea* were prepared to provide distinctive morphological characters.

## Materials and Methods

### Species Identification

Specimens and their genitalia preparations were compared with illustrations of the species provided by Lafontaine (2004) and illustrations of the female type deposited at the Zoological Museum, University of Copenhagen, Copenhagen, Denmark (ZMUC). Dissections of the genitalia were conducted as shown in Dias et al. (2017, 2019) and San Blas et al. (2019).

### Distribution

The distribution map was based on extrapolated label data of specimens deposited at the following collections: CEUCS: Coleção Entomológica da Universidade de Caxias do Sul, Caxias do Sul, Rio Grande do Sul, Brazil; CLAM: Coleção Alfred Moser, São Leopoldo, Rio Grande do Sul, Brazil; DZUP: Coleção Entomológica Padre Jesus Santiago Moure, Curitiba, Paraná, Brazil; Embrapa: Coleção Entomológica da Embrapa Cerrados, Planaltina, Distrito Federal, Brazil; HT: Coleção Hubert Thöny, Camacan, Bahia, Brazil; IOC: Coleção Entomológica do Instituto Oswaldo Cruz, Rio de Janeiro, Brazil; MCTP: Museu de Ciência e Tecnologia da Pontifícia Universidade Católica do Rio Grande do Sul, Porto Alegre, Rio Grande do Sul, Brazil; MZUSP: Museu de Zoologia da Universidade de São Paulo, São Paulo, Brazil; UFPel: Coleção Entomológica do Museu Ceslau Biezanko, Universidade Federal de Pelotas, Pelotas, Rio Grande do Sul, Brazil; VOB: Coleção Vitor Osmar Becker, Camacan, Bahia, Brazil; CNC: Canadian National Collection of Insects, Ottawa, Canada; IFML: Instituto y Fundación Miguel Lillo, Tucumán, Argentina; USNM: National Museum of Natural History, Washington D.C., USA; ZMUC: Zoological Museum, University of Copenhagen, Copenhagen, Denmark, and from the following literature data: Audant (1935), Ferguson (1953), Salinas (1967), Zikán and Zikán (1968), Specht (1972), Valencia and Valdivia (1973), Tarragó et al. (1975), Silveira-Neto et al. (1977), Maes and Tellez (1988), Schotman (1989), Specht and Corseuil (2002), Lafontaine (2004), Specht et al. (2004, 2005), Zagatti et al. (2006), Zenker et al. (2010), Prestes (2014), and Torretta et al. (2009). Maps were prepared using SimpleMappr (Shorthouse 2010). Figured and dissected specimens are deposited at the DZUP.

### Biological Parameters

Biological parameters were obtained under laboratory conditions at the Laboratório de Entomologia, Embrapa Cerrados, Brasília, Distrito Federal, Brazil. Larvae were kept in a controlled rearing room (25 ± 1°C, 70 ± 10% RH, and a 14-h photophase) and were fed on an artificial diet (Montezano et al. 2013a). Females collected in the field were kept individually in cylindrical plastic cages (10 cm Ø and 15 cm high); the tops of the cages were closed with voile and the bottom with Petri dishes (10.5 cm Ø) lined with filter paper. Adults were fed with 10% honey solution. Filter papers with eggs were maintained inside polystyrene containers (11.5 cm × 11.5 cm × 3 cm) with wet paper towel until hatching. The experiment started with 168 larvae that hatched on the same day, obtained from eggs laid by three females collected in the field (54, 64, and 50 larvae from each female).

### Egg

The embryonic survival and incubation period were estimated from the 168 eggs from the three females collected in the field, and 16,883 eggs from females of the first generation of moths reared in laboratory. Because granulate cutworm females usually lay individual eggs, rectangular pieces of craft paper or voile (80mm × 60mm) containing different numbers of eggs were cut out. After the eggs were counted, each piece of paper or voile was placed in a polystyrene container with a moist cotton pad (57 mm Ø) with autoclaved water until hatching.

### Larva

The newly hatched larvae were individually transferred using a fine brush to a white PVC container with transparent plastic cover (38 mm Ø × 27 mm height) 12 h after hatching. Each plastic container has a coin-shaped portion of the artificial diet (25 mm Ø × 5 mm) cut with a stainless-steel cutter. Daily observations were made between 8 and 10 a.m. to verify survival and instar change by a collection of the molted head capsules. Every 48 h, larvae were transferred to new containers with a fresh portion of the artificial diet, which allowed for greater asepsis. The head capsules were individually stored in microcentrifuge tubes labeled by larva and measured with a micrometer under a microscope. In cases where the head capsule and exuviae were not recovered (presumed to have been consumed by the larva), instar changes were noted by comparing the size with other larvae and by the presence of pieces of the head capsules in the fecal pellets. The growth ratio was determined by head capsule size, measuring the distance between genae (mm) of each instar from 50 randomly sampled larvae that did not feed on head capsules (25 females and 25 males). The mean growth ratio was calculated by dividing the mean head capsule width of each instar by the mean head capsule width of the previous instar. The prepupal period is characterized by the interruption of feeding and decrease in size. Larvae that did not feed for 24 h were considered prepupae and were transferred into a transparent plastic container (10 cm Ø × 5cm height) containing autoclaved expanded vermiculite moistened with autoclaved water. The prepupae always built the pupal chamber attached to the bottom of the container, allowing detection when the pupal metamorphosis has occurred.

### Pupa

Pupae were kept in the same container and conditions as in the prepupal period and checked daily to note adult emergence and to maintain moisture with a few drops of autoclaved water. Two days after pupation, the pupae were removed from pupal chambers for sex determination (Madruga et al. 2019) and weighed with a high precision (1 mg) semianalytical scale. Considering that sex determination is only possible during the pupal stage, the identity of each larva was preserved throughout the study, allowing backtracking the development and sex of each individual larvae from hatching to adult.

### Adult

The experiment involving adults used 27 female–male pairs formed with adults that emerged on the same day from the first generation of moths reared in the laboratory. Each pair was maintained within cylindrical plastic containers (10 cm Ø × 15 cm high). The top of the containers was closed with brown voile fastened with a rubber band, facilitating the visualization of eggs (which are white when laid). The bottoms were closed with Petri dishes (10.5 cm Ø). Both the bottom and the walls were lined with craft paper. The roughness of craft paper makes it easier for the moths to stick to the wall and the brown color facilitates the counting of newly laid eggs. Every day, each pair was collected in a glass test tube (2 cm Ø × 20 cm high) and transferred to a newly prepared container. The voile and the craft papers were stored in plastic bags properly identified, and eggs were counted under a stereomicroscope. When it was not possible to count all the eggs on the same day, the plastic bags with the samples were frozen (−17°C) to be counted later. Each container with a pair of moths received daily two Petri dishes (50 mm Ø) filled with cotton wool, one containing an artificial diet and the other with autoclaved mineral water. The artificial diet was prepared with honey (10 g), sorbic acid (1 g), methylparaben (1 g), sucrose (60 g), and distilled water (1,000 ml). All components were dissolved in distilled water and the resulting solution was kept under refrigeration (7°C). To stimulate the feeding of moths, Pilsen beer was daily added to the solution at a proportion of 1:4 beer to the diet and made available to the insects (Hoffmann-Campo et al. 1985). To evaluate the effect of pupal weight on reproductive parameters (Tisdale and Sappington 2001, Specht et al. 2016), records made on the second day after metamorphosis were kept and the fecundity was correlated with pupal weight. Mortality was recorded during the daily changes of moths to new cages; dead moths were kept in 2.5 ml microtubes with ethyl alcohol (96° GL). Dead females were dissected to determine the number of matings by counting the number of spermatophores received during copulation. Fecundity (the number of eggs per female), longevity, and the duration of the preoviposition, postoviposition, and oviposition periods were determined. As the number of pairs in a cage interfere with fertility (Milano et al. 2008, Specht et al. 2016), and as the moths have free access to each other in the field, a cage with five pairs emerged on the same day were kept under the same conditions as the isolated couples mentioned above. Eggs for the study were randomly selected from the 16,883 eggs laid by these couples between the first and last oviposition.

### Host Plants

New records of *F. subterranea* host plants in Brazil were obtained by asystematic surveys conducted from June 2003 to February 2011 in Caxias do Sul, Rio Grande do Sul (by A.S. and D.G.M.), and from June 2013 to August 2017 at the Estação Experimental da Embrapa Cerrados, Planaltina, Distrito Federal, Brazil (by A.S., F.A.D.B., and P.V.M.V.). During these surveys, all larvae found near to the ground feeding on any plant in the field were collected and reared in the laboratory until the emergence of adults. Emerged adults were identified as *F. subterranea* by comparison with type specimens (deposited at ZMUC) and figures provided by Lafontaine (2004). Plants used as hosts by *F. subterranea* were collected and identified by a botanist, Dr. Ronaldo A.Wasum, from the Herbarium of the Universidade de Caxias do Sul by comparison with herbarium specimens and literature. An extensive list of *F. subterranea* host plants was compiled from a variety of databases (Robinson et al. 2010), literature data (Snow and Callahan 1968, Tietz 1972, Heppner 2007, Capinera 2019 [United States], Silva et al. 1968 [Brazil], Maes and Tellez Robleto 1988 [Nicaragua], Posada Ochoa 1989 [Colombia], Coto et al. 1995 [Central America]), and scientific reports (Ingram et al. 1938, Jefferson et al. 1959, Raulston et al. 1972, Wilfret 1980, Smith et al. 1996, Drezner 2014, Gilligan et al. 2019, McCartney et al. 2019). Host plant taxonomy follows the taxonomy used by the Commonwealth Agricultural Bureaux International (CABI 2019), and the United States Department of Agriculture (USDA 2020). Host plants were organized according to the family, genus, species, common name (when available), and references. New records are explicitly indicated.

### Data Analysis

All biological parameters were analyzed using descriptive statistics. The fecundity, longevity of both sexes, and the duration of pre- and oviposition periods were correlated with the number of copulations for each pair: unmated females (*n* = 5 pairs), females that mated once (*n* = 11 pairs), twice (*n* = 8 pairs), and three times (*n* =3 pairs). Shapiro–Wilk was used to confirm normality of data, and Levene’s test to assess the equality of variances. Analysis of Kruskal–Wallis was used to verify the significance of the treatments and χ ^2^ test was used for the comparison of the means at a 5% probability level (α = 0.05). Pearson’s linear correlation method was used to verify possible association between larval duration on pupal weight. Likewise, we assessed whether there was any effect of the pupal weight on fecundity. To verify the significance of the coefficients of the model (linear coefficient and coefficient of determination), a *t*-test was used. To verify the quality of the adjusted model, the coefficient of determination (*R*^2^) was used. All statistical procedures were performed in IBM SPSS Statistics for Windows, version 19 (IBM Corp., Armonk, NY). Biotic potential (BP) was calculated using the equation described in Silveira-Neto et al. (1976). The life table data of age-specific survival (*l*_*x*_) and the number of offspring per day (*m*_*x*_) were graphically presented by plotting the probability of values at the midpoint of each time interval. Using the life table, the values of *F. subterranea* reproductive parameters were calculated. The net reproductive rate (*R*_0_), given by the ratio between the number of females in two successive generations and the mean generation time (*T*), which is the mean number of days from the birth of the parents to the birth of offspring; the intrinsic rate of increase (*r*_*m*_), and the finite rate of increase (λ) were calculated as in Silveira-Neto et al. (1976).

## Results

### Species Identification

All development stages of *F. subterranea* are remarkably similar to several other species of cutworms. However, adults can be distinguished by the male doubly serrated antennae, the small and round orbicular spot and the reniform spot connected by a narrow bar and the translucent pearly white hind wing in both sexes (Figs. 4–5). Additionally, the long and posterior truncated valvae of the male genitalia (Figs 6–7) and the female genitalia (Figs 8–9) are decisive to distinguish the species from all other species in the genus.

### Distribution

The compilation of literature data indicates that *F. subterranea* is widely distributed in the Americas (Fig. 10). The examined material deposited in entomological collections greatly extends the reported distribution of *F. subterranea* in South America, significantly extending its range in Colombia, Ecuador, Peru, all regions of Brazil (with most records in the southeastern and southern regions), and to Argentina, in the provinces of Salta, Tucumán and La Rioja from Northwestern Argentina and Misiones and Santa Fe from Eastern Argentina (Fig. 10, black circles). Records from Córdoba and Buenos Aires, provided by Torretta et al. (2009), and the record for Uruguay, provided by Biezanko et al. (1974), are uncertain and need further confirmation (Fig. 10, gray stars).

### Biological Parameters

**Figure 11.**
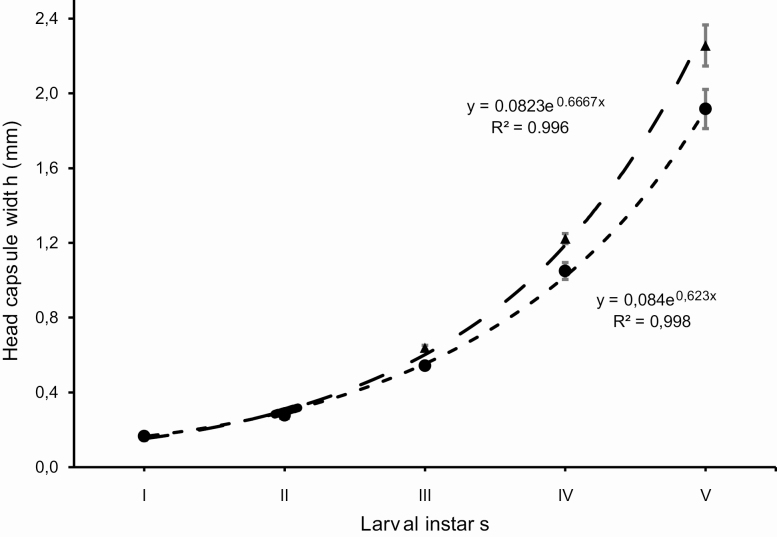
Head capsule sizes of females (triangles and dashed line) and males (circles and dotted line) of *Feltia subterranea* (Fabricius, 1794) instars. Insectsreared under controlled conditions (25 ± 1°C, 70 ± 10% RH, and 14 h photophase) and larvae reared on an artificial diet.

*Feltia subterranea* survival rates are high in all development stages (95.68%) (Table 1). There was low variation in the duration of each stage among individuals, confirmed by the standard deviation and range values (Table 1). Egg, larval, pupal, and adult stages took 6.08%, 44.2%, 27.00%, and 22.73% of the total development time, respectively (Table 1). Therefore, more than three-quarters of the development time corresponds to the immature stages. All larvae developed through five instars, and there were significant differences between sexes for head capsule size (Table 2; Fig. 11), instar, and stage durations for the fourth and fifth instar (Table 3). The growth of the head capsule size between instars forms a geometric progression, with a growth ratio of 1.93 for females and 1.85 for males (Table 2, Fig. 11). The duration of the larval stage is significantly longer in females than in males from the fourth instar on (including prepupae) (Table 3). In contrast, the duration of the pupal stage is significantly shorter in females than in males. However, when larval and pupal stage durations are combined, there are no significant differences on total development time as a function of sex (Table 3).

**Table 1.** Developmental stage survival and duration of *Feltia subterranea* (Fabricius, 1794) reared under controlled conditions (25 ± 1°C, 70 ± 10% RH, and 14 h photophase) on an artificial diet

Stage	N initial–final	Survival (%)	Duration (d)	Range (d)
Egg	16,883–16,649	98.614	3.000 ± 0.000	—
Larval	168–164	97.619	17.458 ± 0.619	17–24
Prepupal	164–164	100.000	4.367 ± 0.543	3–6
Pupal	164–163	99.390	13.331 ± 0.673	12–16
Adult (pairs)	27–	—	11.222 ± 2.361	6–18
Overall	—	95.679	49.378	

**Table 2.** *Feltia subterranea* (Fabricius, 1794) head capsule width (mm) (*n* = 25 for each sex) at each instar and respective growth ratios

Instar	Female		Male		Significance
	Mean ± SE	Growth ratio	Mean ± SE	Growth ratio	
I	0.168 ± 0.022		0.165 ± 0.010		ns
II	0.280 ± 0.020	1.669	0.278 ± 0.014	1.680	ns
III	0.639 ± 0.019	2.279	0.543 ± 0.013	1.955	**
IV	1.223 ± 0.045	1.913	1.050 ± 0.027	1.935	**
V	2.257 ± 0.131	1.845	1.917 ± 0.063	1.826	**
Mean	—	1.927	—	1.849	—

Larvae reared under controlled conditions (25 ± 1°C, 70 ± 10% RH, and 14 h photophase) on an artificial diet. Sig.: Comparisons between means of females and males using a Student *t*-test, considering different variances, at a significance level of 95% (ns—*P* > 0.05; *—*P* < 0.01).

**Table 3.** Mean larval duration (days) and standard deviation (SD) of instars and pupae of *Feltia subterranea* (Fabricius, 1794)

Duration	Females (87)	Males (76)		Both (163)
	Mean ± SD	Mean ± SD	Sig.	Mean ± SD
Larval instar I	3.089 ± 0.286	3.039 ± 0.196	ns	3.066 ± 0.249
Larval instar II	3.011 ± 0.105	3.000 ± 0.000	ns	3.006 ± 0.078
Larval instar III	2.978 ± 0.148	2.987 ± 0.115	ns	2.982 ± 0.134
Larval instar IV	4.189 ± 0.538	4.013 ± 0.258	**	4.108 ± 0.441
Larval instar V (active feeding)	4.444 ± 0.705	4.118 ± 0.325	**	4.295 ± 0.585
PP (prepupae)	4.500 ± 0.604	4.211 ± 0.410	**	4.368 ± 0.543
Total - PP	17.711 ± 0.675	17.158 ± 0.367	**	19.054 ± 2.372
Total + PP	22.211 ± 0.868	21.368 ± 0.512	**	21.825 ± 0.838
Pupae	13.078 ± 0.622	13.632 ± 0.608	**	13.331 ± 0.673
Total larvae + pupae	35.289 ± 1.201	35.000 ± 0.712	ns	35.157 ± 1.015

Insects reared under controlled conditions (25 ± 1°C, 70 ± 10% RH, and 14 h photophase) and larvae reared on an artificial diet.

Comparisons between means of females and males using a Student *t*-test, considering different variances, at a significance level of 95% (Ns—*P* > 0.05; **—*P* < 0.001).

**Figure 12.**
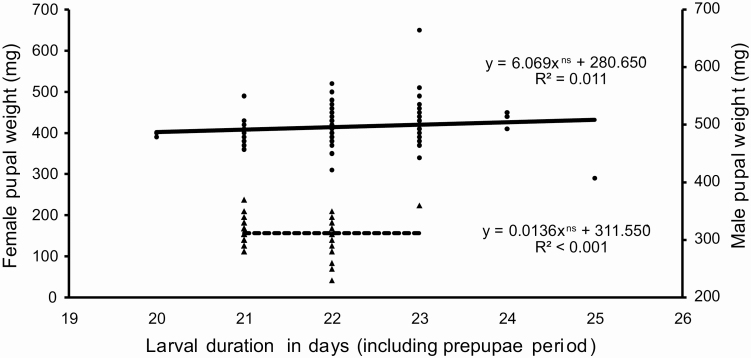
The relation between larval duration (days) and pupal weight (mg) of females (full line and circles) and males (dashed line and triangles) of *Feltia subterranea* (Fabricius, 1794). Insects reared under controlled conditions (25 ± 1°C, 70 ± 10% RH, and 14 h photophase) and larvae reared on an artificial diet.

**Figure 13.**
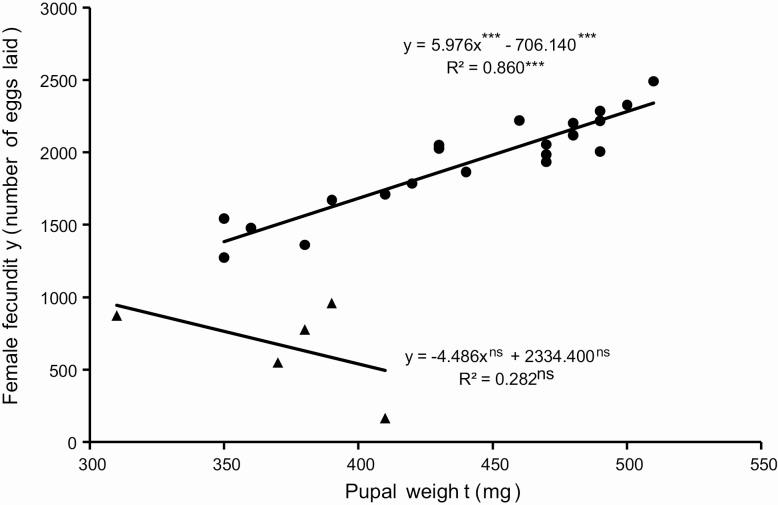
Relation between pupal weight (mg) and fecundity of mated (circles) and unmated (triangles) females of *Feltia subterranea* (Fabricius, 1794). Insects reared under controlled conditions (25 ± 1°C, 70 ± 10% RH, and 14 h photophase) and larvae reared on an artificial diet.

**Figure 14.**
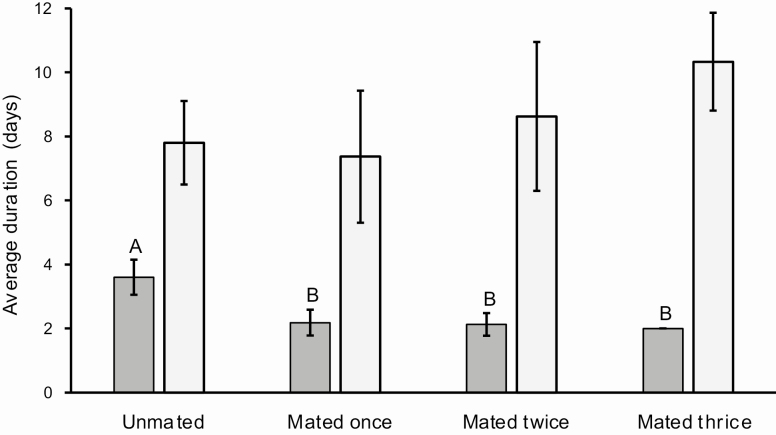
Average and standard deviation of preoviposition (dark gray) and oviposition periods (light gray) of *Feltia subterranea* (Fabricius, 1794) unmated females (*n* = 5) and females that mated once (*n* = 11), twice (*n* = 8), or three times (*n* = 3).

**Figure 15.**
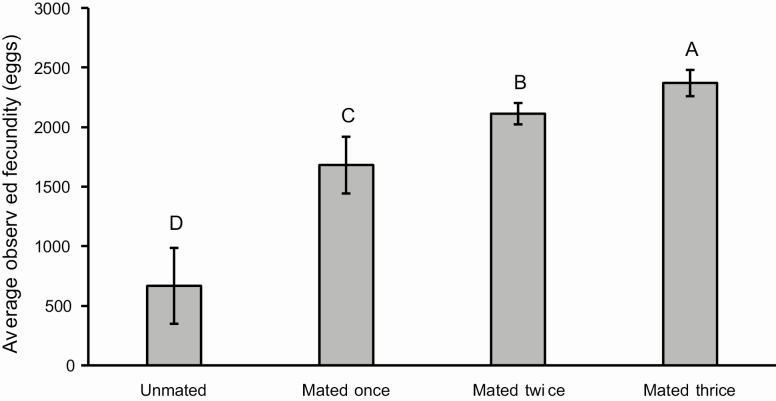
Average and standard deviation observed fecundity of *Feltia subterranea* (Fabricius, 1794) for unmated females (*n* = 5), and females that mated once (*n* = 11), twice (*n* = 8) or three times (*n* = 3).

**Figure 16.**
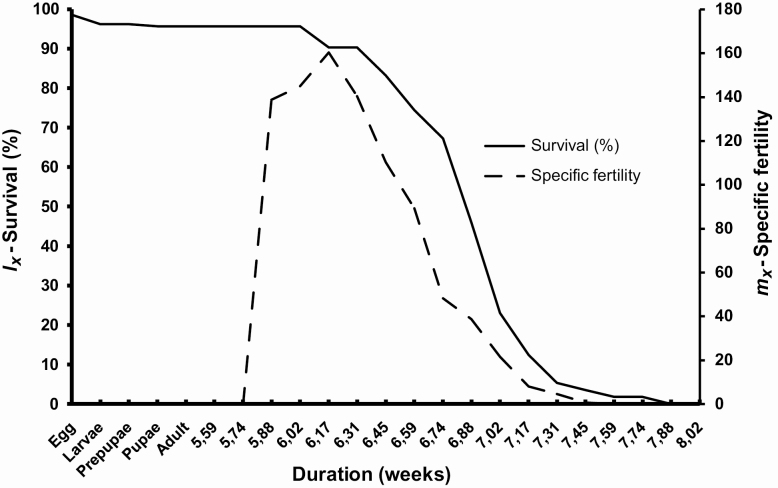
Relationship between age-specific survival (*l*_*_x_*_—full line) and the number of offspring per day (*m*_*_x_*_—dashed line) of *Feltia subterranea* (Fabricius, 1794).

The sex ratio calculated from 163 pupae, 87 females and 76 males, was 0.534, which does not differ significantly from a 1:1 ratio (χ ^2^ = 0.742; *P* = 0.389). The head capsule size (even though there is a large variation, especially in females), and pupal weights are significantly larger in females than in males (Table 4). Regression analysis does not identify a relationship between larval stage duration and pupal weight for males and females (Fig. 12). There is no significant difference between males and females in adult longevity (Table 5). However, some individuals died after the sixth day, while others lived for over 2 wk. Females lay eggs from the third day of the adult stage, laying up to 2,494 eggs. From the female moths kept in pairs (*n* = 27), 5 did not mate, 11 mated once, 8 mated twice, and 3 mated three times. Regression analysis including females that mated at least once indicates a positive correlation between pupal weight and fecundity, which is not observed between unmated females (Fig. 13). Moreover, the preoviposition period of unmated females was significantly longer than females that mated at least once (χ ^2^ = 16.623; df = 3; *P* < 0.001). There was no significant difference (χ ^2^ = 5.295; df = 3; *P* = 0.151) between the oviposition period between unmated females and females that mated one or more times (Fig. 14). Although there is no significant relationship between pupal weight and number of matings (χ ^2^ = 7.300; df= 3; *P* = 0.063), there is a close relationship between the number of matings and the fecundity (χ ^2^ = 23.273; df = 3; *P* < 0.001), i.e., the number of laid eggs by female (Fig. 15). The net reproductive rate (*R*_o_) was 799.983 times per generation, and the mean generation time (*T*) was 43.777 d. The intrinsic rate of increase (*r*_*m*_) was 0.153, with a finite increase rate (λ), meaning that the number of females added to the population per female that will generate another female is 1.165. The maximum rates of population increase occurred between days 42 and 43, during the sixth week of development (Fig. 16). Each female laid, on average, 1,696.963 eggs, with a sex ratio (sr) of 0.534; the overall egg survival is 95.679%, yielding 1,623.638 viable individuals per female (d). The average duration of the life cycle (43.78 d) corresponds to 8.34 generations per year (*n*). Thus, considering the environmental resistance (er) as null, we obtained the following result for the equation BP = (sr * d)^*n*^ – er BP = (0.534 × 1,623.638)^8.338^ – 0 = 3.124 × 10^24^ individuals per female. In other words, each female could generate more than a septillion offspring per year.

**Table 4.** Mean pupal weight (mg) with the number of weighed pupae (n) and standard error (SE) of *Feltia subterranea* (Fabricius, 1794)

Sex	n	Mean ± SE	Range
Female	87	415.444 ± 49.744	291–650
Male	76	311.842 ± 23.364	232–371
Significance		*	-

Insects reared under controlled conditions (25 ± 1°C, 70 ± 10% RH, and 14 h photophase) and larvae reared on an artificial diet. Comparison of means using a Student *t-*test, considering different variances, at a significance level of 95% (*P < 0.001).

**Table 5.** Means, standard deviation (SD) and range of longevity, pre-, post- and oviposition periods and fecundity of 27 couples of *Feltia subterranea* (Fabricius, 1794)

Sex	Biological parameter	Mean ± SD	Range
Female	Longevity (days)	10.889 ± 2.40	6 −15
	Pre-oviposition (days)	2.423 ± 0.694	2–4
	Post-oviposition (days)	0.269 ± 0.555	0–2
	Oviposition (days)	8.077 ± 2.107	4–12
	Fecundity (eggs)	1,696.963 ± 591.874	166–2,494
Male	Longevity (days)	11.556 ± 2.309	6 −18

Insects reared under controlled conditions (25 ± 1°C, 70 ± 10% RH, and 14 h photophase) and larvae reared on an artificial diet. Comparisons of male and female mean longevity using a Student *t* test, considering different variances, at 5% level of significance (ns—*P* = 0.304).

### Host Plants

In total, 159 botanical taxa belonging to 41 families are recorded as *F. subterranea* host plants. The compilation of literature data records 100 taxa being used as hosts, and further 35 and 24 taxa are newly recorded as hosts to the species in Distrito Federal and Rio Grande do Sul, respectively (Table 5). The families with the greatest number species used as hosts are: Fabaceae (22), Poaceae (19), Asteraceae (16), Brassicaceae (13), Solanaceae (12), Amaranthaceae (7), Cucurbitaceae (7), and Malvaceae (5). It is noteworthy the large number of native weeds used as host plants could represent a source of infestation of crops in the agricultural landscape.

## Discussion

### Specific Identity

Misidentification of *F. subterranea* with other species, such as *F. submontana* (Köhler, 1961) in Brazil (Dias et al. 2019), and *F. lutescens* (Blanchard, 1852) and *Pseudoleucania anteposita* (Guenée, 1852) in Argentina and Chile are reported. *Feltia lutescens* and *P. anteposita* were considered synonyms of *F. subterranea*, corresponding to some of the southern most records assigned to the species in the literature. Lafontaine (2004) revised the species, synonyms, and type material of *F. subterranea* and concluded that *F. lutescens* and *P. anteposita* were valid species, and the latter was combined with *Pseudoleucania* Staudinger, 1899. *Feltia lutescens* differs from *F. subterranea* by its deeply serrated antenna, with a double tuft of setae on each serration, and longer and more coiled vesica of aedeagus in males and ductus bursae and apophyses much longer in females. Furthermore, *F. lutescens* is restricted to the extreme south of South America, with its northern most records in Santiago, Chile and Neuquén, Argentina (Jana-Sáenz 1989), farther south than all known records of *F. subterranea*. *Feltia subterranea* is comprehensively distinguished from *F. submontana* by Dias et al. (2019).

### Distribution

*Feltia subterranea* is widely distributed throughout the Americas (Fig. 10), and literature data reveal a strong bias for records in North and Central American countries and the Caribbean islands, with very few records from South America. Records from Córdoba and Buenos Aires, provided by Tarretta et al. (2019), are uncertain. All specimens identified as ‘*F. subterranea*’ from these provinces deposited in the IFML correspond to its former synonym, *F. lutescens* (most similar to the type of *Euxoa bosqi* Köhler, 1945). Similarly, all specimens from Chile identified as ‘*F. subterranea*’ also correspond to *F. lutescens* (most similar to the type of *Noctua lutescens* Blanchard, 1852). Thus, the occurrence of *F. subterranea* in Chile is unlikely. Biezanko et al. (1974) report the occurrence of the species in Uruguay, no specific location given (Fig. 10, gray star). However, Uruguayan specimens of *F. subterranea* were not located at UFPel, where Ceslau Biezanko usually deposited his specimens. The presence of *F. subterranea* in Uruguay cannot be ruled out entirely, as the southern most record for the species is from Pelotas, Rio Grande do Sul, Brazil, which is very close to the Uruguayan border.

### Biological Parameters

*Feltia subterranea* development duration is similar to that observed by Vendramim et al. (1982) using the same temperature conditions with both kale leaves and a similar, but different formulated pinto bean-based artificial diet as larval food. The development duration is also similar to other cutworm species that do not go through diapause, especially regarding the relative durations of each development stage, as *Agrotis ipsilon* (Hufnagel, 1767) (black cutworm) (Bento et al. 2007); *Peridroma saucia* (Hübner, 1808) (variegated cutworm) (Moreno Farjado and Serna Cardona 2006); and *Anicla infecta* (Ochsenheimer, 1816) (green cutworm) (Teston et al. 2001). Preliminary data of pupal recovery from digging in fallow fields in the Florida Panhandle, Jay, FL, which is a transition zone between temperate and tropical areas, indicated no diapause condition and adults emerge approximately in 1 week (S.V.P.M.).

The high survival rate obtained for *F. subterranea* in this study was ¼ superior obtained for a São Paulo population (Vendramim et al. 1982) whose larvae were fed on an artificial diet (61.74%) and kale leaves (60.99%). The survival was also higher than observed for several other owlet moths and cutworms reared in nearly identical conditions by some of the authors (A.S., D.G.M., V.F.R.-S., S.V.P.M., and I.S.B.) (e.g., Montezano et al. 2013a,b, 2014a,b, 2015a,b, 2019a,b; Specht and Roque-Specht 2016, 2019; Silva et al. 2018a, b). Considering that the adequacy of different formulations of owlet moths artificial diets modify the developmental paramenters, (e.g., Bavaresco et al. 2004) the lower survival rates of *F. subterranea* obtained by Vendramim et al. (1982) on artificial diet, may be partly accounted to the differences between the artificial diet formulations used by those authors and in the present study.

### Eggs

The embryonic duration of *F. subterranea* (3 d without variation) are in line with previous studies, such as Snow and Callahan (1968) at room temperature, and Vendramim et al. (1982) at a controlled temperature, with the same average temperature used in this study, but with more variation (±2°C). It is important to note that Lee and Bass (1969) observed a large average embryonic duration of 4.8 d using the same average temperature used in this study. Similarly, Walkden (1950) acknowledged 5 d of embryonic duration in summer generations and 6 d in winter generations. There is no significant variation between embryonic durations of *F. subterranea* (Vendramim et al. 1982) and other owlet moths (Specht et al. 2019) of the same population reared with different diets. This discrepancy in embryonic duration in different studies can be linked to genetic and/or latitudinal variations among different populations (Brito et al. 2019). Studies conducted in North America collected specimens in latitudes over 30°N parallel, while Vendramim et al. (1982) and the current study collected specimens at the 22°S and 15°S parallel, respectively. Owlet moths are generally larger in higher latitudes, and larger moths have longer embryonic durations.

The fertility of *F. subterranea* in this study (Table 1) is considerably higher than in other studies. The average fertility reported by Vendramim et al. (1982) was 76% for larvae fed on kale leaves and 83% for larvae fed on an artificial diet, adults of both treatments fed on a 10% honey solution. The average fertility reported by Snow and Callahan (1968) for larvae that fed on peanut leaves was 49%, ranging from 0% to 89.72%, adult diet was not reported. To maximize mating (see Snow and Callahan 1968, Kehat and Gordon 1975, Ellis and Steele 1982, Rogers and Marti Jr. 1997, Specht et al. 2016), multiple pairs confined in a cage resulted in a higher mating number, observed fecundity, and fertility rate. Moreover, the artificial diet used to feed adults in this study (Hoffmann-Campo et al. 1985) may have positively influenced the observed fecundity and fertility.

### Larvae

In this study, all *F. subterranea* larvae developed through five instars (Tables 2 and 3). Other studies indicate that *F. subterranea* usually develops through six instars. Although Snow and Callahan (1968) indicate that the species develop through five (26%), six (65%), and seven (8%) instars for individuals from the same population, other studies indicate that the species develop through six instars (e.g., Crumb 1929, Walkden 1950, Vendramim et al. 1982, Capinera 2019). It was expected that the marked sexual dimorphism in the size of the larva, pupa (Tables 2 and 3; Fig. 12), and adult (Lafontaine 2004) would cause the larger females to develop through an additional instar, as observed in other Lepidoptera (Esperk and Tammaru 2006, 2010; Esperk et al. 2007a,b), including several other species of owlet moths such as representatives of *Spodoptera* Gueneé, 1852 (Montezano et al. 2013a, 2014b, 2015a; Specht and Roque-Specht 2016).

Vendramim et al. (1982) did not find significant differences in biological parameters (weight, duration, fecundity, etc.) between individuals of *F. subterranea* reared on natural and artificial diet. However, the results of the present study, using a different formulation of artificial diet, but otherwise somewhat similar rearing conditions, indicate a reduction in development duration, reduction in the number of instars, and an increase in fecundity and survival. These differences are most likely related to the artificial diet used in this study. The artificial diet used in this study is probably more appropriate for *F. subterranea* than the artificial diet used by Vendramim et al. (1982). Other studies including cutworms (e.g., Santos and Shields 1998, Esperk et al. 2007b, Cohen 2003, Schneider 2009) indicate that a more appropriate diet is related to faster development durations and a reduction in the number of instars.

The sexual dimorphism of *F. subterranea* was described only for adults of the species (Lafontaine 2004), and differences between sexes of larval stages were widely disregarded (e.g., Walkden 1950, Snow and Callahan 1968, Lee and Bass 1969, Vendramim et al. 1982). Nevertheless, from the third instar on the size of the head capsule is different among sexes (Table 2, Fig. 11), and, from the fourth instar on, the duration of stages is also different (Table 3).

The significantly larger larval stage and prepupal period duration in females corroborates with the hypothesis that larger insects need more time to development to reach the pupal stage (Esperk and Tammaru 2006, 2010, Esperk et al. 2007b). However, the relation between larval duration and pupal weight of each sex separately demonstrates that larval stage duration is not related to the pupal weight (Fig. 12). Thus, the hypothesis that larger pupal weight is related to larger development duration only holds between individuals of different sexes, but not between individuals of the same sex.

The head capsule growth rate of *F. subterranea* (1.8) (Table 2) is larger than the growth rate of 1.5 ± 2 estimated by Dyar (1890) for Lepidoptera. The higher growth rate in *F. subterranea* may be related to instars and to the morphology of the cutworms (Agrotini), in which the head capsule is retractable and unusually small in relation to the body size. For example, the average head capsule size of the last instar (fifth) of *F. subterranea* is 2.26 mm and the pupal weight is 415.44 mg, while in *S. frugiperda* (J.E. Smith, 1797) (growth ratio = 1.52) reared in similar conditions, the average head capsule size of the last instar (sixth) is 2.80 mm and the pupal weight is only 230.53 mg (Montezano et al. 2019a). This indicates that the head capsule growth rate is linked not only to effects listed by Esperk et al. (2007a), but also to the morphology associated with feeding habits and relationships with the environment, such as the subterranean habits of most cutworms.

### Pupae

The average pupal weight of *F. subterranea* (0.368 ± 0.065mg) was similar to the weight measured by Vendramim et al. (1982). As expected, there is a significant difference (of about 100 mg) between the average weight of the male and female pupae (Table 4). The difference is probably a manifestation of the sexual dimorphism (Lafontaine 2004) observed in some Noctuinae, including in species of *Feltia* (Specht et al. 2013, Dias et al. 2017, 2019, San Blas and Agrain 2017). It is interesting to note the variation in pupal weight between individuals, especially females, where the heaviest pupa is twice the weight of the lightest one (Table 4). This variation was not expected since the individuals were reared under identical conditions. However, major weight variations were also observed in species of *Spodoptera* reared under similar conditions (Montezano et al. 2013a, 2014b, 2015a, 2019a; Specht and Roque-Specht 2016).

It is interesting to note the significantly longer pupal stage duration of males in relation to females (Table 3), also reported to other species of Noctuinae (Montezano et al. 2013a, 2014b, 2015a, 2019a; Specht and Roque-Specht 2016). The longer duration of the pupal stage in males balanced the longer duration of the larval stage in females, and therefore, the emergence of the males and females happened at roughly the same time. Even though several studies report both protogyny and protandry in Lepidoptera (see Degen et al. 2015), the simultaneous emergence of males and females of *F. subterranea* was similarly reported by Cline and Habeck (1977) in Florida, USA.

### Adult

The different number of matings (unmated, *n* = 5; mated once, *n* = 11; mated twice, *n* = 8; mated three times, *n* = 3) is similar to that observed by Snow and Callahan (1968) and Cline and Habeck (1977). The majority of females will mate at least once, to a maximum of four matings under laboratory conditions (Snow and Callahan 1968). The oviposition period of *F. subterranea*, starting in the third night after adult emergence, is similar to previous observations (Cline and Habeck 1977) and is also significantly linked to matings (Fig.14). The longer preoviposition period for unmated females is related to the necessity of fertilization to stimulate the beginning of the oviposition period, as previously observed in *F. subterranea* (Cline and Habeck 1977) and other species of Noctuidae (Montezano et al. 2013b, 2014a, 2015b, 2019b; Specht and Roque-Specht 2019). Conversely, in the present study, the oviposition period was similar among unmated females and females that mated once, twice, or three times (Fig. 14). In other studies, the oviposition period lasted longer for unmated females (Montezano et al. 2013b, 2014a, 2015b, 2019b; Specht and Roque-Specht 2019). The fecundity was much larger in the present study (Table 5) than other *F. subterranea* studies (Jones (1918): 529.4 laid eggs and 264.8 eggs retained on the abdomen of dissected females, 794.2 eggs in total (average of 10 moths); Crumb (1929): 403 eggs (one moth), and 325 eggs (average of three moths); Walkden (1950): 970 eggs retained on the abdomen of a dissected female; Snow and Callahan (1968): 1,142 eggs (average of nine moths), Lee and Bass (1969): 647 eggs; Cline and Habeck (1977): 746 eggs (average of mated females) and 286 eggs (average of unmated females); Vendramim et al. (1982): 1,035.15 eggs (average of individuals fed on natural diet as a larvae) and 1,390.30 eggs (average of individuals fed on artificial diet as a larvae, adults fed on 10% honey solution). The dissimilarity may be related to the adequacy of the larval diet (Scheider 2009), pupal weights, and/or the adequacy of the adult diet. However, the high fecundity of *F. subterranea* in the present study is similar to the fecundity of other species of cutworms that can lay an average of 2,000 eggs (e.g., Archer et al. 1980, Bento et al. 2007, Specht et al. 2013).

The fecundity of mated females is higher than the fecundity of unmated females, as previously observed by Cline and Habeck (1977) (Figs. 13 and 15). While the fecundity of unmated females is unrelated to pupal weights, the fecundity of mated females is significantly related to pupal weights (Fig. 13). Larger pupae produce females with higher fecundity, as observed in *S. eridania* (Cramer, 1782) (Specht et al. 2016) and *S. frugiperda* (Montezano et al. 2019b). This observation is directly related to the greater investment in female size to maximize fecundity (Tammaru et al. 2002). Although there is no significant relationship between the number of matings and pupal weight in the present study (χ ^2^ = 7.300; df = 3; *P* = 0.063), the increase in the number of matings significantly increases the fecundity of *F. subterranea* (Fig. 15). The significant positive relationship between fecundity and number of matings is observed in other species of owlet moths (e.g., Snow et al. 1970; Ellis and Steele 1982; Chu and Yang 1991; Rogers and Marti Jr 1994, 1996; Ward and Landolt 1995; Landolt 1997; Hou and Sheng 1999; Sadek and Anderson 2007; Montezano et al. 2013b, 2014a, 2015b, 2019b; Specht and Roque-Specht 2019). Thus, several factors may be related to these observations, such as the specific vigor of the individuals used in the experiment, hormonal effects in multiple mating females (Zeng et al. 1997), and the availability of nutrimental material obtained from spermatophores in ‘re-mating’ received by females during mating in some Lepidoptera (but not in Noctuidae) (Boggs and Watt 1981, Greenfield 1982).

### Life Table

The life table parameters presented for *F. subterranea* are similar to other polyphagous owlet moth pests that fed on an artificial diet (Barrionuevo et al. 2012; Montezano et al. 2013a, 2014b, 2015b, 2019b; Silva et al. 2018a,b; Specht and Roque-Specht 2019) or on their preferred host plants (Greenberg et al. 2001, Santos et al. 2005, Farahani et al. 2011, Bortoli et al. 2012, Specht et al. 2019). These parameters are characterized by a high net reproductive rate (*R*_o_) and short mean generation time (*T*). The other high reproductive parameters values (intrinsic rate of increase [*r*_*m*_] and finite rate of increase [λ]) are as a function of *R*_0_ and *T* combined with a high survival. For example, some pest species of *Spodoptera*, such as *S. albula* (Walker, 1857) and *S. eridania*, reared under similar controlled conditions, present relatively lower *R*_0_ values (353.90 and 560.53) linked to similar low *T* values (37.19 and 35.81 d). Conversely, *S. cosmioides* (Walker, 1858) and *S. dolichos* (Fabricius, 1794) present higher *R*_0_ values (1,711.98 and 2,191.77) linked to similarly higher *T* values (46.41 and 56.19 d). In these cases, all *r*_*m*_ and λ values ranged between 0.135 and 0.177 and 1.13 and 1.18, respectively. *Spodoptera frugiperda*, the most important pest species of the genus, presents higher *R*_0_ value (1,079.73) linked to lower *T* value (32.00), resulting in markedly higher *r*_*m*_ (0.22) and λ (1.24) values. Therefore, it is important to note that the reproductive parameters presented here for *F. subterranea* (*R*_0_ = 799.983; *T* = 43.777; *r*_*m*_ = 0.153 and λ = 1.165) indicate that under favorable conditions the species has the potential to increase its population rapidly, owing to the species and individual polyphagy, potentially causing crop injury and damage at the beginning of the growing season. This potential is further supported by many cases in which human involvement was required to protect crops from losses caused by outbreaks of *F. subterranea* (Jones 1918, Crumb 1929, Chamberlin and Madden 1942, Walkden 1950, Snow and Callahan 1968, Lee and Bass 1969, Morgan and French 1971, Adlerz 1975, Bass and Johnson 1978).

### Biotic Potential

The biotic potential of *F. subterranea* (3.124 × 10^24^ individuals per female) is particularly high, and similar to the biotic potential of some major pest species of owlet moths and loopers (Specht et al. 2019), *Helicoverpa armigera* (Hübner) (old world cotton bollworm) (Silva et al. 2018a) and species of *Spodoptera* (e.g., Montezano et al. 2013a, 2014b, 2019b).

The association of *F. subterranea* high biotic potential, high larval polyphagy, wide distribution, and high dispersal capacity may explain the reports of outbreaks of the species in North America (e.g., Cook and Horne 1905, Jones 1918, Chamberlin and Madden 1942), Central America (Cook and Horne 1905, Wolcott 1941, Maes and Tellez Robleto 1988, Coto et al. 1995, Saunders et al. 1998) and South America (Mariconi 1954, Costa 1959, Salinas 1967, Vendramim et al. 1982, Posada Ochoa 1989) in the past. *Feltia subterranea* occurs simultaneously with other species of cutworms throughout its range; its populations are usually larger in warmer climates (Cook and Horne 1905, Jones 1918, Audant 1935, Wolcott 1948, Salinas 1967, Snow and Callahan 1968, Bass and Johnson 1978, Maes and Tellez Robleto 1988, Posada Ochoa 1989, Coto et al. 1995, Saunders et al. 1998) and smaller in colder climates (Jones 1918, Walkden and Whelan 1942, Snow and Callahan 1968, Specht 1972, Tarragó et al. 1975, Bass and Johnson 1978, Specht and Corseuil 2002, Zenker et al. 2010) in relation to other cutworm species.

### Host Plants

The record of 159 plants from 41 families reported in this study (Table 6) represents a contribution to the list of *F. subterranea* host plants, but possibly represents just a fraction of the number of plants used as hosts. The great majority of host plants recorded in the literature are linked to economic crops; the relatively small number of weeds usually corresponds to species most commonly found in agricultural ecosystems, possibly playing a role as a source of infestation of this species in the landscape. Besides the economic crops and weeds in cultivated systems listed among the 59 new records of host plants in this study, plant species in natural systems are also included. Similar to other species of agricultural importance, such as the black cutworm (Crumb 1929; Rings et al. 1975; Busching and Turpin 1976, 1977; Costa and Link 1984; Link and Costa 1984; Link and Pedrolo 1987) and the variegated cutworm (Crumb 1929, Rings et al. 1976), *F. subterranea* is highly polyphagous, both as a species (i.e., larvae of the species are able to feed in several species of plants) and as individuals (i.e., an individual larvae are able to feed in several species throughout its development) (Crumb 1929, Chamberlin and Madden 1942, Link and Knies 1973, Santos and Nakano 1982, Link and Costa 1984, Leonard et al. 1993, Allen et al. 2018). The host plant range of this species indicates its potential to establish and cause outbreaks in cover crops and conservation-tillage systems (e.g., Oliver and Chapin 1981, Gaylor and Foster 1987, Leonard et al. 1993, Allen et al. 2018).

**Table 6.** Host plants of *Feltia subterranea* (Fabricius, 1794) compiled from the literature, with new records from Brazil

	Family	Scientific name and authority	Common name	References
1.	Alismataceae	*Echinodorus grandiflorus* (Cham. & Schltdl.) Micheli.	Chapéu-de-couro	**
2.	Amaranthaceae	*Amaranthus* sp.		7, 14
3.		*Amaranthus cruentus* L.	Red amaranth	*
4.		*Amaranthus deflexus* L.	Large fruit amaranth	*
5.		*Amaranthus dubius* Mart. ex Thell.	Spleen amaranth	*
6.		*Amaranthus hybridus* L.	Slim amaranth	*
7.		*Amaranthus spinosus* L.	Spiny amaranth	5, 16
8.		*Celosia cristata* L.	Cockscomb	*
9.	Amaryllidaceae	*Allium cepa* L.	Onion	3, 5,9, 11, 12, 16
10.		*Allium sativum* L.	Garlic	3, 9, 11
11.		*Coriandrum sativum* L.	Coriander	**
12.	Apiaceae	*Apium graveolens* L.	Wild celery	5, 16, 17
13.		*Daucus carota* L.	Carrot	5, 9, 11,13,14,16
14.		*Petroselinum crispum* (Mill.) Nyman ex A.W. Hill	Parsley	13
15.	Aquifoliaceae	*Ilex crenata* Thunb.	Japanese holly	13
16.	Araceae	*Caladium* sp.	Caladium	13
17.	Asparagaceae	*Asparagus officinalis* L.	Asparagus	7, 11,13, 14
18.	Asteraceae	*Arctium lappa* L.	Greater burdock	7,13, 14
19.		*Bidens pilosa* L.	Beggar-ticks	*
20.		*Calendulaofficinalis* L.	Pot marigold	**
21.		*Chrysanthemum morifolium* Ramat	Florist’s daisy	**
22.		*Cichorium endivia* L.	Endive	13
23.		*Conyza bonariensis* (L.) Cronquist	Asthmaweed	*
24.		*Dahlia pinnata* Cav.	Margarita	**
25.		*Gerbera jamesonii* Bolus ex Hook. F.	Barberton daisy	**
26.		*Helianthus annuus* L.	Sunflower	11, 14
27.		*Lactuca sativa* L.	Lettuce	4, 5,7, 9, 11, 13, 14, 16
28.		*Lactuca serriola* L.	Prickly lettuce	14
29.		*Senecio brasiliensis* (Spreng.) Less.	Brazilian ragwort	*
30.		*Stevia rebaudiana* (Bertoni) Bertoni	Candyleaf	*
31.		*Tagetes* L.	*marigold*	13
32.		*Taraxacum officinale* F.H. Wigg.	Dandelion	5, 7, 13, 14
33.		*Xanthium strumarium* L. var. *canadense* (Mill.) Torr. & A. Gray	Canada cocklebur	5, 7, 13, 14
34.	Boraginaceae	*Symphytum officinale* L.	Common comfrey	**
35.	Brassicaceae	*Brassica napus* L. var. *napus* L.	Rape	**
36.		*Brassica oleracea* var. *acephala* DC.	Kale	5,16
37.		*Brassica oleracea* var. *botrytis* L.	Cauliflower	3, 5, 16
38.		*Brassica oleracea* var. *capitata* L.	Cabbage	3, 5, 7, 11, 9, 16
39.		*Brassica oleracea gemmifera DC*	Brussels sprouts	5, 71316
40.		*Brassica oleracea* var. italica Plenk	Broccoli	5, 7, 1316
41.		*Brassica rapa* L. var. *rapa* L.	Turnip	5, 713, 14, 16
42.		*Brassica rapa L.* var. *amplexicaulis* Tanaka & Ono	Field mustard	3
43.		*Capsella bursa-pastoris* (L.) Medik.	Shepherd’s purse	5, 713, 14, 16
44.		*Eruca vesicaria* (L.) Cav. ssp. *sativa* (Mill.) Thell.	Rocket salad	*
45.		*Lepidium sativum* L.	Gardencress pepperweed	*
46.		*Lepidium virginicum* L.	Virginia pepperweed	7, 13, 14
47.		*Raphanus sativus* L.	Cultivated radish	5, 13,16
48.	Cactaceae	*Carnegiea gigantea* (Engelm.) Britton & Rose	Saguaro	15
49.		*Hylocereus undatus* (Haw.) Britton & Rose	Pitaya	*
50.	Caryophyllaceae	*Dianthus caryophyllus* L.	Carnation	12
51.		*Gypsophila paniculata* L.	Baby’s breath	6
52.	Chenopodiaceae	*Chenopodium quinoa* Willd.	Quinoa	18
53.		*Beta vulgaris* L. ssp. *cicla* (L.) W.D.J. Koch	Chard	3
54.		*Beta vulgaris* ssp. *vulgaris* var. *conditiva* Alef.	Beet	3, 5, 7, 11, 9, 13, 14, 16
55.		*Beta vulgaris* var. *saccharifera* Alef.	Sugar beet	*
56.		*Spinacia oleracea* L.	Spinach	5, 16
57.	Commelinaceae	*Commelina diffusa* Burm. F.	Climbing dayflower	**
58.		*Tradescantia zebrina* hort. ex Bosse	Inch plant	**
59.	Convolvulaceae	*Dichondra* J.R. Forst. & G. Forst.	Ponysfoot	2, 5
60.		*Ipomoea batatas* (L.) Lam.	Sweet potato	5, 7,9,11, 13, 14,16
61.		*Ipomoea purpurea* (L.) Roth	Tall morning-glory	*
62.	Cucurbitaceae	*Citrullus lanatus* (Thunb.) Matsum. & Nakai var. *lanatus*	Watermelon	3, 45, 9, 11, 14, 16
63.		*Cucumis melo* L.	Melon	3, 4, 9, 10, 11,13, 14
64.		*Cucumis sativus* L.	Cucumber	3, 9, 11, 14
65.		*Cucurbita moschata* Duchesne var. *toonas* (Makino) Makino	Kabocha	11
66.		*Cucurbita pepo* L.	Marrow	3, 9
67.		*Fevillea cordifolia* L.	Antidote vine	*
68.		*Sechium edule* (Jacq.) Sw.	Chayote	9, 11
69.	Euphorbiaceae	*Chamaesyce prostrata* (Aiton) Small	Prostrate sandmat	**
70.		*Codiaeum variegatum* (L.) A. Juss.	Garden croton	*
71.		*Manihot esculenta* Crantz	Cassava	9, 11
72.		*Ricinus communis* L.	Castor bean	*
73.	Fabaceae	*Acacia mearnsii* De Willd.	Black wattle	4, 14
74.		*Arachis hypogaea* L.	Peanut	5, 11, 12, 13,1416
75.		*Arachis pintoi* Krapov. & Gregory	Pinto peanut	*
76.		*Cajanus cajan* (L.) Millsp.	Pigeonpea	*
77.		*Cicer arietinum* L.	Chickpea	11
78.		*Crotalaria breviflora* DC.	Short flower rattlebox	*
79.		*Desmodium adscendens* (Sw.) DC.	Zarzabacoa galana	*
80.		*Desmodium tortuosum* (Sw.) DC.	Dixie ticktrefoil	5
81.		*Glycine max* (L.) Merr.	Soybean	5, 9, 16
82.		*Lathyrus latifolius* L.	Perennial pea	5
83.		*Lespedeza* Michx.	Lespedeza	5, 16
84.		*Medicago lupulina* L.	Black medick	5,
85.		*Medicago sativa* L.	Alfalfa	5, 13, 14,16
86.		*Mucuna pruriens* var. *utilis* (Wall. ex Wight) Baker ex Burck	Velvet bean	14
87.		*Phaseolus lunatus* L.	Sieva bean	13, 14
88.		*Phaseolus vulgaris* L.	Kidney bean	3, 45, 7, 9, 10, 11, 12, 13, 14, 16
89.		*Pisum sativum* L.	Pea	5, 7, 11, 12, 13, 14,16
90.		*Pueraria montana* (Lour.) Merr.	Kudzu	*
91.		*Trifolium* sp.	Clover	5, 7, 13,14,16
92.		*Trifolium repens* L.	White clover	
93.		*Vicia faba* L.	Fava bean	5, 11, 16
94.		*Vigna unguiculata* (L.) Walp	Cow pea	5, 9, 11, 13, 14, 16
95.	Geraniaceae	*Geranium traversii* Hook. f	Cranesbill	**
96.	Iridaceae	*Cipura campanulata* Ravenna	Cipura	5
97.		*Gladiolus* sp.	Gladiolus	8
98.	Lamiaceae	*Melissa officinalis* L.	Bee balm	**
99.		*Mentha spicata L.*	*Spearmint*	**
100.		*Ocimum basilicum* L.	Sweet basil	**
101.		*Origanum majorana* L.	Sweet marjoram	**
102.	Lauraceae	*Persea americana* Mill	Avocado	5, 11, 13
103.	Liliaceae	*Lilium candidum* L.	Madonna lily	**
104.	Malvaceae	*Abelmoschus esculentus* (L.) Moench	Chimbinvoy	4, 14
105.		*Gossypium barbadense* L.	Creole cotton	14
106.		*Gossypium hirsutum* L.	Cotton	4, 5, 7, 9, 11, 12,13, 14,16
107.		*Hibiscus cannabinus* L.	Bimli-jute	13
108.		*Sida rhombifolia* Linn.	Arrow-leaf	*
109.	Onagraceae	*Fuchsia regia* (Vand Ex Vell) Munz	Fuchsia	**
110.		*Ludwigia peruviana* (L.) H. Hara	Peruvian primrose-willow	*
111.	Oxalidaceae	*Oxalis articulata* Savigny	Azedinha	**
112.	Passifloraceae	*Passiflora incarnata* L.	Purple passionflower	5, 7,13, 14,16
113.	Pedaliaceae	*Sesamum indicum* L.	Sesame	9, 11
114.	Piperaceae	*Piper* sp.	Pepper	5, 13
115.	Plantaginaceae	*Plantago* sp.	Plantain	5, 7, 13, 14,16
116.	Poaceae	*Avena sativa* L.	Cultivated oat	4, 11, 14
117.		*Cynodon dactylon* (L.) Pers.	Bermuda grass	2, 5
118.		*Cynodon nlemfuensis* Vanderyst	African Bermuda grass	*
119.		*Digitaria sanguinalis* (L.) Scop.	Crabgrass	*
120.		*Eremochloa ophiuroides* (Munro) Hack.	Centipede grass	13
121.		*Hordeum vulgare* L.	Barley	5
122.		*Lolium perene* L. ssp. *multiflorum* Lam. Husnot	Annual ryegrass	2
123.		*Oryza sativa* L.	Rice	4, 9, 10, 11,14
124.		*Panicum miliaceum* L.	Proso millet	*
125.		*Paspalum distichum* L.	Knotgrass	5
126.		*Pennisetum purpureum* Schum.	Elephant grass	*
127.		*Saccharum officinarum* L.	Sugarcane	1, 9
128.		*Sorghum bicolor* L. Moench ssp. *bicolor*	Grain sorghum	9,16
129.		*Stenotaphrum secundatum* (Walter) Kuntze	St. Augustine grass	5, 13
130.		*Triticum aestivum* L.	Wheat	4,5, 7, 13, 14,16
131.		*Urochloa brizantha* (Hochst. Ex A. Rich. R. Webster	Palisade grass	*
132.		*Urochloa maxima* (Jacq.) R. Webster	Guinea grass	*
133.		*Urochloa plantaginea* (Link) R. Webster	Plantain signalgrass	*
134.		Zea mays L.	Corn	4,5, 7, 9, 11, 12, 13,14,16
135.	Polygoniaceae	*Polygonum aviculare* L.	Prostrate knotweed	7, 13,14
136.		*Rumex crispus* Linn	Curled dock	**
137.	Portulacaceae	*Portulaca grandiflora* Hook.	Rose moss	*
138.	Phytolaccaceae	*Phytolacca dodecandra* L’Her.	Pokeweed	*
139.	Rosaceae	*Fragaria ananassa* Duchesne	Cultivated strawberry	5, 11,12, 16
140.		*Prunus* sp.	Peach	5, 13,16
141.	Rubiaceae	*Coffea arabica* L.	Coffee	9, 11,14
142.	Rutaceae	*Citrus* sp.	Citrus	4,11,14
143.		*Ruta graveolens L.*	Common rue	**
144.	Solanaceae	*Capsicum annuum* L.	Bell pepper	3, 5, 7, 1113, 14, 16
145.		*Nicandra physalodes* (L.) Scop.	Apple of Peru	5, 7,13,14
146.		*Nicotiana tabacum* L.	Tobacco	4,5, 7,9, 10, 11, 13,14,16
147.		*Petunia* sp.	Petunia	13
148.		*Physalis peruviana* L.	Peruvian groundcherry	*
149.		*Physalis philadelphica* Lam.	Mexican groundcherry	17
150.		*Solanum aethiopicum*. L	Jiló	4,14
151.		*Solanum lycopersicum* Mill	Tomato	4,5, 7,9,11, 13, 14,16
152.		*Solanum melongena* L	Eggplant	3, 4,5,7, 9, 13,14,16
153.		*Solanum seaforthianum* Andrews	Brazilian nightshade	14
154.		*Solanum sisymbriifolium* Lam.	Sticky nightshade	**
155.		*Solanum tuberosum* L.	Potato	4,5, 7, 9, 11, 12, 13,14,16
156.	Tropaeolaceae	*Tropaeolum majus* L.	Nasturtium	*
157.	Verbenaceae	*Lantana camara* L.	Lantana	**
158.		*Phyla nodiflora* (L.) Greene	Turkey tangle fogfruit	13
159.	Violaceae	*Viola tricolor* L.	Johnny jumpup	**

**References**: 1. Ingram et al. (1938), 2. Jefferson et al. (1959), 3. Salinas (1967), 4. Silva et al. (1968), 5. Snow and Callahan (1968), 6. Raulston et al. (1972), 7. Tietz (1972), 8. Wilfret (1980), 9. Maes and Tellez (1988), 10. Posada Ochoa (1989), 11. Coto et al. (1995), 12. Smith et al. (1996), 13. Heppner (2007), 14. Robinson et al. (2010), 15. Drezner (2014), 16. Capinera (2019), 17. Gilligan et al. (2019), 18. McCartney et al. (2019). New records to Brazil: Distrito Federal (*) and Rio Grande do Sul State (**).

*Feltia subterranea* larvae feed on debris and can survive without food sources for weeks, and disperse in search of suitable host plants (Crumb 1929, Chamberlin and Madden 1942). This larval behavior is relevant to IPM, since the application of herbicides should be considered 4 to 6 wk before planting the crop to prevent outbreaks of this pest (Leonard et al. 1993). In cases where postemergence weed control with herbicides is not possible, pyrethroid insecticide application in a narrow band behind the planter is recommended (Allen et al. 2018).

The available data about the immature stages of cutworm species (e.g., Specht 1972, Link and Kies 1973, Angulo and Weigert 1975, Bryan et al. 2000, Baudino 2004, Corró Molas et al. 2017) and adults collected by light traps (e.g., Hills 1968, Tarragó et al. 1975, Lara et al. 1977, Lara and Silveira Neto 1977, Silveira Neto et al. 1977, Specht and Corseuil 2002, Specht et al. 2005, Zenker et al. 2010, Bernardi et al. 2011) indicate spatial and temporal variations of the species as a function of climate conditions, available crops, and insect management. Therefore, a local assessment of cutworm species and their host plants is recommended to avoid yield loss, particularly due to the stand reduction. The proper management of *F. subterranea* should consider soil and weed management strategies (Leonard et al. 1993) and natural biological control, such as the preservation of natural enemies, thus reducing operational costs and preserving the environment and protecting human health. Previous studies list several natural enemy organisms of cutworms, including *F.subterranea*. These organisms include microorganisms (Jones 1918, Crumb 1929, Seaver and Waterston 1946, Adlerz 1975, Hamm and Lynch 1982, Hamm et al. 1986), predators and parasitoids (Jones 1918, Crumb 1929, Sauer 1947, Lima 1949, Arnaud 1957, Bravo 1958, Silva et al. 1968, Adlerz 1975, Guimarães 1977, Saunders et al. 1998, Fernandes et al. 2014, Amiune and Valverde 2017, Capinera 2019), bats (Dood et al. 2015, Pinzari et al. 2019), and birds (Genung and Green Jr. 1974). Supplementary long-term studies should be conducted in the field to assess the importance of these organisms to the population dynamics of *F. subterranea* under natural conditions (see Baudino 2005, Pereira et al. 2018). *Feltia subterranea* also likely plays a role as a pollinator of native plants and commercial crops (Torretta et al. 2009, Benning 2015), and are a food source for other invertebrate and vertebrate animals in natural, anthropized, and agricultural ecosystems (Jones 1918, Crumb 1929, Arnaud 1957, Silva et al. 1968, Genung and Green Jr. 1974, Adlertz 1975, Hamm and Lynch 1982, Hamm et al. 1986, Saunders et al. 1998, Baudino 2005, Dood et al. 2015, Pinzari et al. 2019). *Feltia subterranea* eggs and first instars are present in natural systems, on weeds, and other covers (e.g., Crumb 1929, Chamberlin and Madden 1942, Link and Knies 1973, Santos and Nakano 1982, Link and Costa 1984, Leonard et al. 1993, Allen et al. 2018), and share several natural enemies with others owlet moths (e.g., Silva et al. 1968). The presence of *F. subterranea* may be important to maintain natural biological control in cultivated systems, especially off-season, when the preferred hosts of other owlet moths are not available or when the conditions for its development are limited by other edaphoclimatic factors.
